# Maryland Clinical Society

**Published:** 1895-06

**Authors:** H. O. Reik

**Affiliations:** Secretary, No. 525 N. Howard St., Baltimore, Md.


### Society Reports.

### MARYLAND CLINICAL SOCIETY.

Stated meeting of the Maryland Clinical Society, held March 29th.

In the absence of the president, Dr. James McShane was elected president *pro tem.*

Dr. E. H. Bernstein read a paper on "Ear Complications in LaGrippe."

"If the experience of other aurists has been as mine, this present epidemic has been unusually prolific of serious secondary diseases in the ear, some of which have assumed very curious types, and with me one I had rarely seen before. It is a secondary mastoiditis and purulent otitis media. Ordinarily there are two classes of cases, the one presenting symptoms of naso-pharyngeal catarrh; the other, representing the disease itself, localized in the ear. It is a subdivision of this latter which calls for some remarks; the symptoms simulating acute gastric catarrh to such a degreethat only after several days of suffering would the aural complications be brought to light. The chief characteristic of ear-symptoms in influenza contrasted with other acute infectious diseases is the intense hyperæmia entirely disproportionate to the conditions usually seen. This congestion is also the indirect cause of the complications observed; first, because in the weakened walls of the blood vessels it tends to rupture and hemorrhage; second, because it lights up afresh any inflammation already healed; and third, it renders the mucous membrane highly susceptible to the reception of any other conveyors of disease. Otitis media acuta suppurativa generally begins with sticking, tearing or boring pains in the ear, spreading out over the frontal and occipital region. In children the pain is more intense than in adults, though in the latter it seems almost unbearable. Usually the pain exacerbates towards night, and in the morning the patient may become quiet and sleep several hours; either bodily or mental exertion increases pain. In very intense inflammation slight conjunctivitis, œdema of the lids and photophobia are present before the rupture of the drum. High fever, nausea, unconsciousness and convulsions are concomitants and of such a degree as often to lead one to suspect meningitis or beginning exanthemata with cerebral symptoms. Should we neglect to examine the ear often we are only made cognizant of the aural character of the malady by the purulent discharge when the violent character of the illness subsides, showing the cerebral symptoms to have been caused by acute otitis media. When the mastoid is involved we have great pain and tenderness upon pressure, and the posterior and superior walls of the meatus are hyperæmic. The peculiar set of symptoms to which I wish to call your attention will be best shown by the history of a typical case. The patient has had an attack of la grippe more or less severe. During the height of the disease or while convalescing, typical symptoms of acute gastric catarrh supervene. Headache, nausea, foul breath, furred tongue and great discomfort. Pain in the head and stomach are mutually severe and exacerbate with approach of night. After several of such days, the ear begins to discharge and patient mends. The attention is directed to the ears and great tenderness over the mastoid and tragus are found. The case may now progress favorably as regards pain or the latter, after a short remission, may again become severe and we then have a regular mastoiditis. As to the treatment, I need hardly say that when this is directed to the ear the gastric symptoms promptly give way to medicines which were before powerless. The usual treatment for acute otitis media is to be followed. I should call attention, however, to careful syringing. The all-rubber bulb syringe is far preferable to the piston syringe. I have also found hydrogen dioxide of great help in cleansing the ear, as it gets into portions of the tympanic cavity unattainable by syringing, unless you use the Hartman's canula. In mastoiditis I make use of cold applications or an ointment of belladonna, camphor and mercury and only use Wilde's incision when the inflammation does not succumb to these remedies."

DR. HIRAM WOODS: I think it does not matter so much what syringe we use as how carefully it is used. One of the best methods for cleansing the ear with a small perforation is to put peroxide of hydrogen into the ear, the patient lying with the affected ear up and having him swallow while the mouth and nose are held closed. In this way the solution is forced into the tympanic cavity and thus passes on down into the throat. As regards the doctor's remarks about the use of cold in mastoid cases, I have frequently seen good results therefrom, but my experience with Wilde's incision is so satisfactory and it is such an easy operation, I rely upon it a great deal, and when the patient will consent, it is my favorite way of treating mastoid complications. My experience does not lead me to think that the statement made is correct that children suffer more acutely than adults. Some of the most severe suffering I have ever seen was in cases of otitis media in adults. As regards the cases occurring after this present epidemic of grippe, I have seen many cases of acute aural catarrh, and the principal feature that impressed me was the rapidity with which the tympanum perforated after the first onset of symptoms and the marked and persistent amount of deafness as compared with the evidences of local inflammation. I have also seen two or three cases of inflammation of the external ear as the sequel of grippe, one of which was that of a young lady nineteen years of age, who had just recovered from what was supposed to be grippe. Her first trouble appeared as an abcess on the cheek, this was followed by another on the left side of the nose, and a week later she appeared with a dermatitis of the left side of the face, protrusion of the auricle, swelling over of the mastoid, but suffering little pain. The temperature never went above 100, pulse varied between 80 and 92. Her left eye was entirely closed by œdema of the lids, and the mastoid was tender, especially towards the upper part. Wilde's incision was performed but no pus was found. This condition of œdema of the cheek persisted for several days, and finally pus appeared in the mastoid cut, well up over the pinna. While this was going on, she developed œdema of the right side of the face, with the occurrence of two or three furuncles in the right external ear. I instituted constitutional treatment of iron strychnia and salines and the patient is now practically well. At the hospital I have seen two or three cases resembling this of diffuse otitis externa and showing no middleear disease at all. These cases all gave a history of having had so-called grippe.

DR. HARLAN: In the cases of otitis media said to be the sequel of grippe I have seen nothing peculiar that was referable to the previous disease, and they were all to be treated as ordinary otitis media. Dr. Bernstein spoke of the application of cold for the relief of pain, but said nothing about the use of heat. I have found that even more satisfactory, and usually use it in the form of hot poultices or hot water injections into the ear.

Dr. Edward M. Schaeffer read a paper on "Sanitariums."

Meeting then adjourned.

H. O. REIK, M. D., Secretary,
No. 525 N. Howard St.,
Baltimore, Md.

